# Two Novel Motifs of *Watermelon Silver Mottle Virus* NSs Protein Are Responsible for RNA Silencing Suppression and Pathogenicity

**DOI:** 10.1371/journal.pone.0126161

**Published:** 2015-05-20

**Authors:** Chung-Hao Huang, Weng-Rong Hsiao, Ching-Wen Huang, Kuan-Chun Chen, Shih-Shun Lin, Tsung-Chi Chen, Joseph A. J. Raja, Hui-Wen Wu, Shyi-Dong Yeh

**Affiliations:** 1 Department of Plant Pathology, National Chung Hsing University, Taichung, 40227, Taiwan; 2 Institute of Biotechnology, National Taiwan University, Taipei 106, Taiwan; 3 Department of Biotechnology, Asia University, Wufeng, Taichung, 41354, Taiwan; 4 NCHU-UCD Plant and Food Biotechnology Center, National Chung Hsing University, Taichung 40227, Taiwan; The University of Texas Medical Branch, UNITED STATES

## Abstract

The NSs protein of *Watermelon silver mottle virus* (WSMoV) is the RNA silencing suppressor and pathogenicity determinant. In this study, serial deletion and point-mutation mutagenesis of conserved regions (CR) of NSs protein were performed, and the silencing suppression function was analyzed through agroinfiltration in *Nicotiana benthamiana* plants. We found two amino acid (aa) residues, H113 and Y398, are novel functional residues for RNA silencing suppression. Our further analyses demonstrated that H113 at the common epitope (CE) (^109^KFTMHNQ^117^), which is highly conserved in Asia type tospoviruses, and the benzene ring of Y398 at the C-terminal *β*-sheet motif (^397^IYFL^400^) affect NSs mRNA stability and protein stability, respectively, and are thus critical for NSs RNA silencing suppression. Additionally, protein expression of other six deleted (ΔCR1-ΔCR6) and five point-mutated (Y15A, Y27A, G180A, R181A and R212A) mutants were hampered and their silencing suppression ability was abolished. The accumulation of the mutant mRNAs and proteins, except Y398A, could be rescued or enhanced by co-infiltration with potyviral suppressor HC-Pro. When assayed with the attenuated *Zucchini yellow mosaic virus* vector in squash plants, the recombinants carrying individual seven point-mutated NSs proteins displayed symptoms much milder than the recombinant carrying the wild type NSs protein, suggesting that these aa residues also affect viral pathogenicity by suppressing the host silencing mechanism.

## Introduction

In plants, post-transcriptional gene silencing (PTGS) is an important defense mechanism at RNA level against pathogens. During plant virus infection, the replicative dsRNA intermediates or viral RNAs-induced dsRNAs, synthesized by RNA-dependent RNA polymerase 6 (RDR6) [1] and suppressor of gene silencing 3 (SGS3) [2–4], trigger PTGS in host cells [5,6]. In the RNA silencing pathway, firstly, Dicer-like proteins (DCLs) cleave long double stranded (ds) RNA into 21–24 nts small interfering (si) RNAs [7]. Then, the ds siRNAs are loaded to RNA-induced silencing complex (RISC), and unwound to single-stranded (ss) siRNAs [8–10]. A key component of RISC, the agronaute (AGO) protein, contains two major domains of PAZ and PIWI for RNA binding and endonuclease activity, respectively. Consequently, the siRNA-loaded RISC targets RNA with sequence homology [11] to generate cleavage or block translation for down-regulating gene expression.

Plant virus has a mechanism to counteract the host PTGS, using a RNA silencing suppressor (RSS) to antagonize the host defensive reaction. HC-Pro and 2b proteins are the first identified RSSs [12–14] and they are also viral pathogenicity factors [15,16]. Subsequently, many RSSs have been reported, which disrupt RNA silencing pathway by different mechanisms [17,18] or interacting with other host factors that are essential for gene silencing or silencing suppression [19,20]. Several studies have shown that viral RSSs also interfere with host endogenous microRNA (miRNA) pathways and cause plant abnormal development, such as leaf distortion and limited expansion by early aging [21–24]. Thus, RSSs generally play important roles for viral pathogenicity.

Thrips-borne tospoviruses are classified into Asia type and Euro-America type based on their nucleocapsid protein (NP) sequences and geographical distributions [25–27]. *Watermelon silver mottle virus* (WSMoV), a species of the genus *Tospovirus*, is rampant in Asian countries and causes severe damages on many economically important crops, including cucumber, melon, and watermelon [28–31]. The WSMoV enveloped particles contain tripartite L (large), M (medium) and S (small) genomic RNAs [32–34]. The 3.5 kb ambisense S RNA encodes a nonstructural (NSs) protein and a nucleocapsid protein (NP) in opposite strands. In plant cells, the NSs protein forms filament inclusion body in cytoplasm and responsible for virus virulence [35]. NSs protein blocks RNA silencing pathway by binding to long dsRNA and siRNA [36–38] and has NTPase and 5’-phosphorylase activities *in vitro* [39]. In addition, NSs protein is the avirulence factor (avr) for the resistant gene *Tsw* in pepper [40,41]. Recently, the NSs protein is considered essential for persistent infection and transmission by thrips [42]. Thus, NSs protein has diverse functions in virus replication, transmission, symptom severity and RSS function.

Functional motifs of the NSs proteins of *Tomato spotted wilt virus* (TSWV), the type species of the genus *Tospovirus* and Euro-America type tospoviruses have been previously demonstrated [39,43,44]. N-terminal domain of the NSs protein of TSWV is important for RNA silencing suppression and avirulence [43]. Two highly conserved motifs (^181^GKT^183^ and ^412^YL^413^) of TSWV NSs protein are critical for RSS function [44]. In groundnut bud necrosis virus, the mutation K189A in Walker motif A (GXXXGKT) of NSs protein affects its ATPase activity and mutation D159A in Walker motif B (DEXX) results in partial loss of ATPase and 5’ phosphatase activities [39].

Previously, we produced a monoclonal antibody (MAb) against WSMoV NSs protein, and found that this MAb recognizes the NSs proteins of WSMoV and most Asia type tospoviruses [45]. A common epitope (CE) in WSMoV NSs protein has been identified as a domain of amino acids (aa) 98–120, denoted as NSscon [45]. Recently, our laboratory has minimized NSscon sequence to 9 amino acids (aa 109–117), denoted as nss [46]. This highly conserved common epitope of NSs protein may be responsible for important functions during virus infection, such as RSS function and pathogenicity.

So far, infectious clones for reverse genetics of tospoviruses are still not available. Thus, we used an attenuated heterologous viral vector, *Zucchini yellow mosaic virus* (ZYMV) [47], to analyze the role of WSMoV NSs protein for pathogenicity. In this study, NSs protein with highly conserved motif mutated were analyzed for RSS function. Viral pathogenicity of particular point-mutated NSs mutants was further examined on squash plants, using the ZYMV mild strain vector.

Our results indicated that all conserved regions of NSs protein are critical for RSS function. Moreover, we demonstrate that five aa residues of Asia-type tospoviral WSMoV NSs, which correspond to the RSS functional residues of Europe-America type tospoviral TSWV NSs protein, are critical for RSS function [43,44]. In addition, we found that two novel functional aa residues, H113 at the CE (^109^KFTMHNQ^117^) and Y398 at the C-terminal *β*-sheet motif (^397^IYFL^400^) of NSs protein, are responsible for RSS function. Furthermore, our results indicate that the above aa residues of NSs protein are also responsible for viral pathogenicity on squash plants.

## Materials and Methods

### Plants and virus source

The isolate of *Watermelon silver mottle virus* (WSMoV) was collected from the leaf tissue of diseased watermelon in Changhua, Taiwan and was mechanically inoculated on single lesion host, *Chenopodium quinoa* Willd. (*C*. *quinoa*). After three transfer on *C*. *quinoa*, the virus was maintained in plants of *Nicotiana benthamiana* Domin. by mechanical transfer [30] and subsequently characterized [29]. The isolate of ZYMV, designated TW-TN3, was collected from naturally infected sponge gourd (*Luffa cylindrical* Roem.) in Tainan, Taiwan and also isolated by mechanical transfer on single lesion host, *C*. *quinoa* and subsequently characterized [48]. ZYMV was maintained in squash plants (*Cucurbita pepo* L. var. Zucchini). A full-length cDNA infectious clone, p35SZYMVGFPhis-3 or p35SZYMVGAC were engineered previously [47]. All the plants were grown and maintained at a temperature controlled (25± 3°C) greenhouse.

### Sequence analysis

Sixteen tospoviral NSs protein sequences downloaded from GeneBank were aligned using Vector NTI Version 10 (Invitrogen, Carlsbad, CA, USA), including Calla lily chlorotic spot virus (CCSV, AY867502), Capsicum chlorosis virus (CaCV, DQ256123), *Groundnut ringspot virus* (GRSV, L12048), *Impatiens necrotic spot virus* (INSV, NC_003624), *Iris yellow spot virus* (IYSV, AF001387), Melon severe mosaic virus (MSMV, EU275149), Melon yellow spot virus (MYSV, AB453909), Peanut bud necrosis virus (PBNV, PBU27809), Peanut chlorotic fan-spot virus (PCFSV, AF080526), Peanut yellow spot virus (PYSV, AF013994), *Polygonum ringspot virus* (PoRSV, EF445397), *Tomato spotted wilt virus* (TSWV, ABI94070), Tomato yellow ring virus (TYRV, AY686718), Tomato zonate spot virus (TZSV, EF552433), *Watermelon bud necrosis virus* (WBNV, ABY79094) and *Watermelon silver mottle virus* (WSMoV, AY864852).

### Construction of WT and mutations on conserved regions of NSs for analyzing RNA silencing suppression function

Agroinfiltration expression system was used to analyze RNA silencing suppression of WSMoV NSs protein. The gene-specific primer details used in the study for the constructs are listed in S1 Table. The WSMoV NSs gene was amplified by reverse transcription-polymerase chain reaction (RT-PCR) with the gene-specific primers from the total RNA of WSMoV-infected *N*. *benthamiana* plants. The amplified fragment was cloned in pENTR/D vector (Invitrogen, Carlsbad, CA, USA) to generate pENTR/WNSs, and transferred to pBCo-DC-myc [21] by LR Clonase (Invitrogen, Carlsbad, CA, USA) to generate the binary vector pBCo-WSMoV/NSs. The pBCo-TSWV/NSs was constructed following the same strategy.

**Fig 1:**
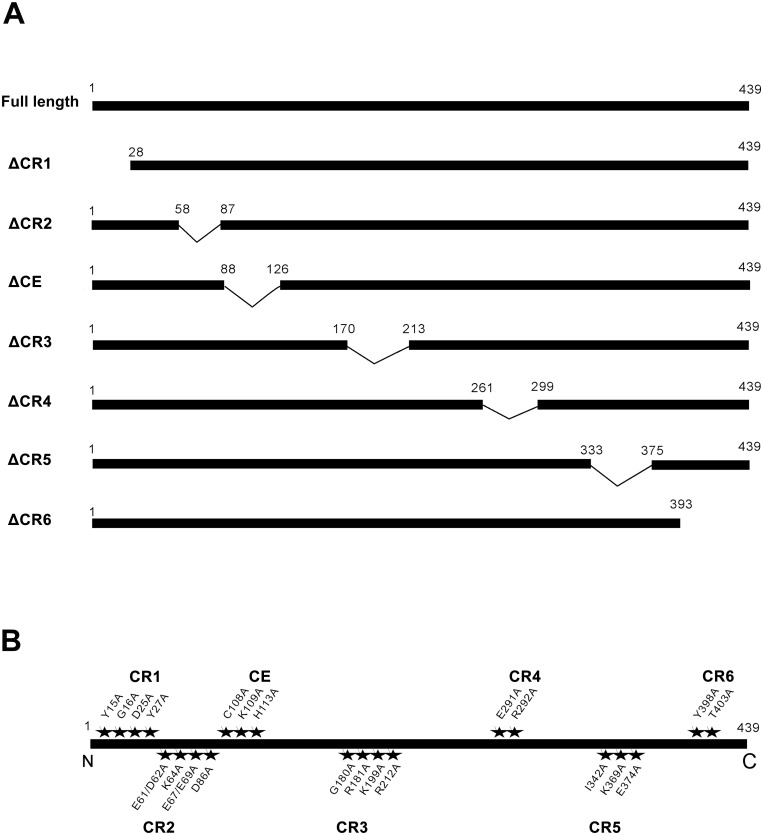
Mutated NSs proteins for analyzing RNA silencing suppression function. **(A)** The maps of different mutants with individual deletions of the highly conserved regions (ΔCR1-ΔCR 6) or the common epitope NSscon (ΔCE) of the NSs protein of *Watermelon silver mottle virus* (WSMoV). The aa positions of the individual deleted regions are indicated. **(B)** The aa positions locating on each conserved region chosen for alanine-mutagenesis in this study.

Deletions on NSs protein, ΔCR1, ΔCR2, ΔCR3, ΔCR4, ΔCR5, ΔCR6 and ΔCE (Fig 1A) were made by assembly PCR on pBCo-NSs, using primer pairs (S1 Table) for introducing the deletions on pBCo-WSMoV/NSs to generate each pBCo- WSMoV/NSs deletion.

Specific mutations on NSs protein, Y15A, G16A, D25A, E61A/D62A, K64A, E67A/E69A, D86A, C108A, K109A, H113A, G180A, R181A, K199A, R212A, E291A, R292A, I342A, K369A, E374A, Y398A and T403A (Fig 1B) and the other mutations, Y398D, Y398E, Y398F, Y398S and Y398T were made by QuikChange XLSite-directed Mutagenesis Kit (Stratagene Corporation, CA, USA) on pBCo-WSMoV/NSs, using primer pairs (S1 Table) for inducing single or double amino acid-substitution mutations on pBCo- WSMoV/NSs to generate each pBCo- WSMoV/NSs mutation. The other RNA silencing suppressor in the binary vector, pBCo-TuMV-HC-Pro, and RNA silencing inducer vector, pBA-GFi, were provided by Shih-Shun Lin (National Taiwan University, Taipei, Taiwan).

### Agroinfiltration and GFP fluorescence analysis

RNA silencing suppression function of deleted or point-mutated NSs proteins was assayed on plants of *N*. *benthamiana* by agroinflitration. An overnight culture of *A*. *tumefaciens* strain ABI containing individual deleted or point-mutated NSs genes was diluted 50-fold in LB medium with 10 mM MES (4-Morpholineethanesulfonic acid) and 0.5 mM acetosyringone. The culture was grown at 28°C up to an optical density of 1.0 at 600 nm absorption. The cells were resuspended in 0.01 M MgCl_2_ with 1 mM acetosyringone and incubated at room temperature for three hours before agroinfiltration which was conducted as previously described [49].

RNA silencing suppression activity assay in leaves of *N*. *benthamiana* plants was performed by co-infiltrating a mixture containing each of mutated NSs, GFP expresser (pBA-GFP) and GFP silencer (pBA-GFi). The silencing suppression activities were recorded by observation of GFP expression at 4 dpa. Visual observation of GFP fluorescence in the infiltrated leaves was performed using a 100 W hand-held long-wave (λ = 395nm) UV lamp (Black Ray model B 100AP, UV Products, Upland, CA). The infiltrated leaves were photographed by a Nikon D70 camera (Nikon, JP) with Cokin 3 and 6 filters (Cokin, Rue des Solets, Silic 457, 94953 Rungis, France).

### Northern blotting

The accumulation of mRNA of NSs, GFP or barstar^R^ (bastaR gene is contained within binary vector, pBCo [21], used as an internal control in agroinfiltration assay) was analyzed at 4 days after agroinfiltration (dpa). Total RNA isolated from 0.05 g infiltrated or inoculated leaves by TRizol reagent (Invitrogen, Carlsbad, CA, USA) was used for northern hybridization analysis. Gene-specific α-^32^P-labeled probes were synthesized by Prime-it II random primer labeling kit (Stratagene, La Jolla, CA, USA) using the WSMoV NSs, GFP, barstar^R^ or 18S rRNA coding region as a template. The rRNA and tRNA were used as loading controls on ethidium bromide (EtBr) stained gels.

### Western blotting

Total soluble proteins from 0.05 g infiltrated or inoculated leaves were extracted by grinding the tissues in 3X protein sample buffer (3% SDS, 30% glycerol, 0.01% bromophenol blue, and 15% *β*-mercaptoethanol.). The samples were incubated at 100°C for 5 min and then the supernatant was collected after centrifugation (8000x*g*, 3 min). The proteins were resolved on a 12% sodium dodecyl sulphate-polyacrylamide gel and transferred to Polyscreen PVDF membrane (PerkinElmer, Waltham, MA, USA). Membranes were probed using corresponding antibodies, i.e., monoclonal (MAb) and polyclonal antibodies (PAb) to WSMoV NSs [45]; PAb to ZYMV HC-Pro [47], ZYMV coat protein (CP) [50] or GFP [51]. Membrane was stained with Coomassie Brilliant Blue R250 and amount of the large subunit of ribulose-1,5-bisphosphate carboxylase/oxygenase (RuBisCO) was used as a loading control. Banding signal intensity was quantified by Kodak Image system 4000MM (EASTMAN Kodak Company, Rochester, NY, USA).

### Construction of NSs mutants for self-interaction analyzed by Yeast two hybrid

To analyze NSs self-interaction by yeast two hybrid assay, the NSs gene was amplified by PCR from pBCo-WSMoV/NSs with gene-specific primer pairs, and then cloned into pGBKT7 and pGADT7 to generate pGBKT7-NSs and pGADT7-NSs, respectively. The other NSs mutation fragments were individually constructed into pGBKT7 and pGADT7, respectively to generate each pGBKT7 and pGADT7-NSs mutations by the same procedure, respectively. The paired plasmids were co-transformed into *Saccharomyces cerevisiae* (*S*. *cerevisiae*) strain AH109 (Clontech, Palo Alto, CA, USA) separately. Transformants were grown at 30°C for 2 days on the synthetic defined (SD) medium lacking leucine (Leu) and tryptophan (Trp), then individual colonies were picked from plate to liquid SD medium lacking Leu and Trp and cultured at 30°C for overnight. Then, liquid yeast culture was transferred to the SD medium lacking histidine (His), Leu and Trp with different concentrations of 3-aminotriazole (3-AT) agar plate to analyze the strength of interaction.

### Construction of NSs gene in ZYMV viral vector for analyzing pathogenicity

In order to construct ZYMV viral vector as a Gateway destination vector for simpler and faster cloning, p35SZAC-DC-nGFP was constructed (S1 Fig). The NSs ORF was amplified by PCR with the gene-specific primers (S1 Table) from pBCo-WSMoV/NSs. The amplified fragment was cloned in pENTR/D vector to generate pENTR/WNSsnonstop. The pENTR/WNSsnonstop was transferred to p35SZAC-DC-nGFP by LR Clonase to generate p35SZAC-NSs-GFP. The other mutated NSs variants were cloned in p35SZAC-DC-nGFP by the same procedure.

## Results

### Identification of highly conserved regions of tospoviral NSs proteins and construction of deleted or point-mutated mutants

Seven highly conserved regions of ten Asia type tospovirus species, identified as conserved regions CR1-6 and the common epitope NSscon (CE), were chosen to create seven different deletion mutants of ΔCR1-ΔCR6 and ΔCE (Fig 1A), respectively, for analyzing their roles in the RSS function. Among the conserved amino acids, the sulfur-containing (C), charged (K, R, D, and E) or phosphorylation-related (Y, T and S) amino acids, including Y15, E61/D62, K64, E67/E69, D86, C108, R181, E291, R292, K369 and E374 of WSMoV NSs protein were chosen for mutagenesis to analyze their effects (Fig 1B). In addition, alignment of the NSs proteins of 16 Asia type and Euro-America type tospovirus species also revealed eleven highly conserved individual amino acids, G16, D25, Y27, K109, H113, G180, K199, R212, I342, Y398 and T403, which are present in the CR1-6 or CE (Fig 1B). Accordingly, single or double point-mutated mutants located at each conserved region were created by alanine (A) substitutions (Fig 1B).

Overall, seven deleted mutants (Fig 1A) and 22 point-mutated WSMoV NSs proteins (Fig 1B) were created and analyzed for their RSS function.

### Proteins of deleted NSs mutants are not detectable and their silencing suppression function is abolished

**Fig 2:**
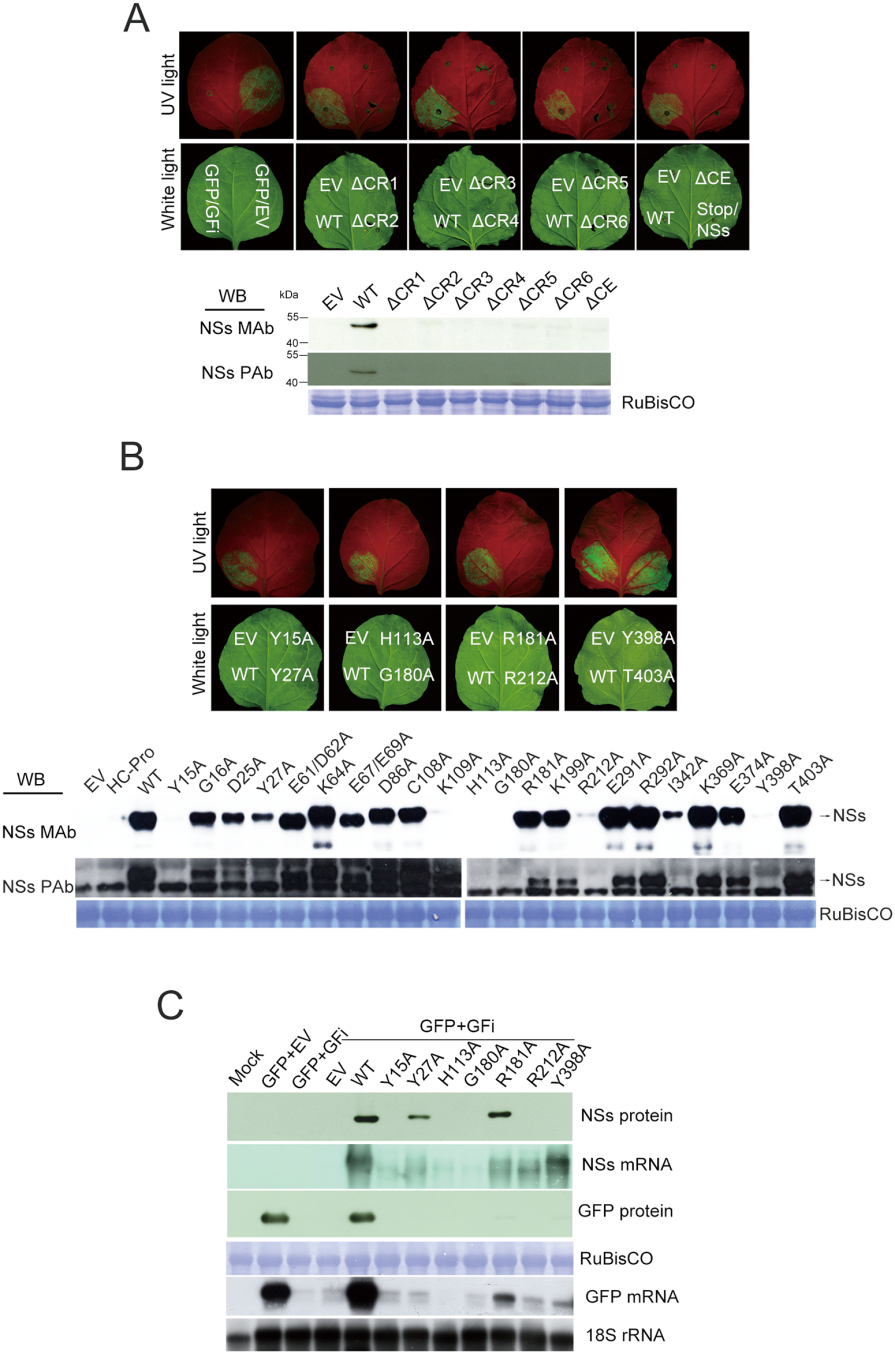
Analysis of RNA silencing suppression function of individually deleted or point-mutated NSs proteins by agroinfiltration in *N*. *benthamiana* plants. The leaf areas agroinfiltrated with each deleted **(A, upper panel)** or point-mutated **(B, upper panel)** NSs constructs were examined under UV and white light. The GFP fluorescence was recorded at 4 days after co-infiltration (dpa) of *Agrobacterium* separately carrying pBA-GFP (the expresser), pBA-GFi (the silencing inducer) and individual constructs with deleted or point mutated NSs proteins described in Fig 1A and 1B. Western blotting was performed for the detection of individually deleted **(A, lower panel)** or point-mutated **(B, lower panel)** NSs proteins expressed at 4 dpa. NSs monoclonal (MAb) and NSs polyclonal antibodies (PAb) were used for detecting NSs protein or NSs protein in which the common epitope was mutated, respectively. Expression levels of point-mutated NSs proteins, NSs mRNA, GFP and GFP mRNA were detected at 4 dpa **(C)**. Coomassie blue-stained RuBisCO protein was used as loading controls for proteins and 18S rRNA used as loading controls for RNAs.

In initial experiments, we analyzed the RSS function of NSs protein by co-infiltrating *A*. *tumefaciens* carrying the wild type (WT) or individual NSs deletion constructs with the two agrobacteria separately carrying a GFP expression construct or a hairpin GFP silence inducer construct (pBA-GFi) into leaf tissues of *N*. *benthamiana* plants. Strong GFP fluorescence was observed in the leaf tissues infiltrated with the vector carrying the WT NSs gene. In contrast, no GFP fluorescence was noticed in leaf tissues infiltrated with the empty vector (EV) and each deletion mutant of ΔCR1-ΔCR6 and ΔCE (Fig 2A, upper panel), indicating that these mutants lost their RSS function.

All the NSs deletion proteins were not detected by anti-NSs MAb, which recognizes the common epitope NSscon. Because the protein of ΔCE does not contain the common epitope, anti-NSs polycolonal antibody (PAb) [45] was used to confirm the absence of the ΔCE NSs protein (Fig 2A, lower panel).

Our data indicated that all the deleted NSs proteins are not detectable in infiltrated leaf tissues of *N*. *benthamiana* plants and their RSS function is abolished.

### Point mutation of critical aa residues of individual conserved regions

Point-mutations for critical aa residues of individual deletion regions (Fig 1B) were further performed and their effects on RSS function were analyzed. The leaf tissues infiltrated with constructs carrying the individual NSs mutants of Y15A, Y27A, H113A, G180A, R181A, R212A or Y398A did not show fluorescence (Fig 2B, upper panel), suggesting that they lost the RSS function. Moreover, the proteins of mutants Y15A, H113A, G180A, R212A or Y398A were not accumulated at detectable levels. Again, since H113A mutation is located within the common epitope NSscon, anti-NSs PAb was used to confirm its absence (Fig 2B, lower panel). In contrast, the leaf tissues infiltrated with other NSs point mutants showed strong GFP fluorescence similar to that of T403A mutant (Table 1, Fig 2B, upper panel), indicating that these mutated residues did not affect RSS function of NSs protein.

**Table 1:** The expression and RNA silencing suppression capability of the mutated NSs proteins of *Watermelon silver mottle virus*, with modifications in conserved aa residues in the highly conserved (CR1-6) regions or the common epitope (CE), analyzed by agroinfiltration in leaf tissues of *N*. *benthamiana* plants.

NSs mutated protein^a^	Conserved region (CR)	Protein expression	RNA silencing suppression
Wild type		+	+
Y15A	CR1	-	-
**Y27A**	CR1	+	-
**H113A**	CE	-	-
**G180A**	CR3	-	-
R181A	CR3	+	-
**R212A**	CR3	-	-
**Y398A**	CR6	-	-
**G16A**	CR1	+	+
**D25A**	CR1	+	+
E61A/D62A	CR1	+	+
K64A	CR2	+	+
E67A/E69A	CR2	+	+
D86A	CR2	+	+
C108A	CE	+	+
**K109A**	CE	+	+
**K199A**	CR4	+	+
E291A	CR4	+	+
R292A	CR4	+	+
**I342A**	CR4	+	+
K369A	CR5	+	+
E374A	CR5	+	+
**T403A**	CR6	+	+

^a^ Bold proteins indicate the aa residues are highly conserved in NSs proteins of all tospoviruses of Asia and Euro-America types from databases. The others are only from Asia type tospoviruses.

High levels of GFP mRNA accumulation were found in leaf tissues infiltrated WT NSs constructs, showing strong intensity of GFP fluorescence. Of the seven mutated NSs proteins, which lost their RSS function, only proteins of Y27A and R181A were accumulated at detectable levels that are lower than that of WT (Fig 2C), whereas Y398A protein was not detected. The accumulation of Y27A, R181A and Y398A mRNAs was detectable, but at much lower levels than that of WT (Fig 2C). The accumulation of GFP mRNA and protein in leaf tissues infiltrated with R181A and Y398A was detectable, but much lower than that of the WT (Fig 2C). In contrast to Y27A and R181A, the Y398A protein was not detected, but the accumulation of NSs mRNA in leaf infiltrated with Y398A was higher than that of other mutants. This result implies that the dysfunction of silencing suppression activity of Y398A may be due to protein instability, not directly due to the impairment of its functional motif.

Taken together, our data firstly indicate that deletion of the NSscon epitope (ΔCE) and H113A mutation located at the CE affects NSs protein accumulation and completely abolishes the RSS function. Secondly, the other deletion (ΔCR1-ΔCR6) and point-mutants (Y15A, Y27A, G180A, R181A, R212A and Y398A) also diminish the capability of RSS function. Thirdly, among all mutants only Y27A and R181A proteins were expressed at detectable levels. Fourthly, Y398A protein may be degraded at post-translational level.

### Undetectable deleted or point-mutated NSs proteins can be rescued by potyviral HC-Pro

**Fig 3:**
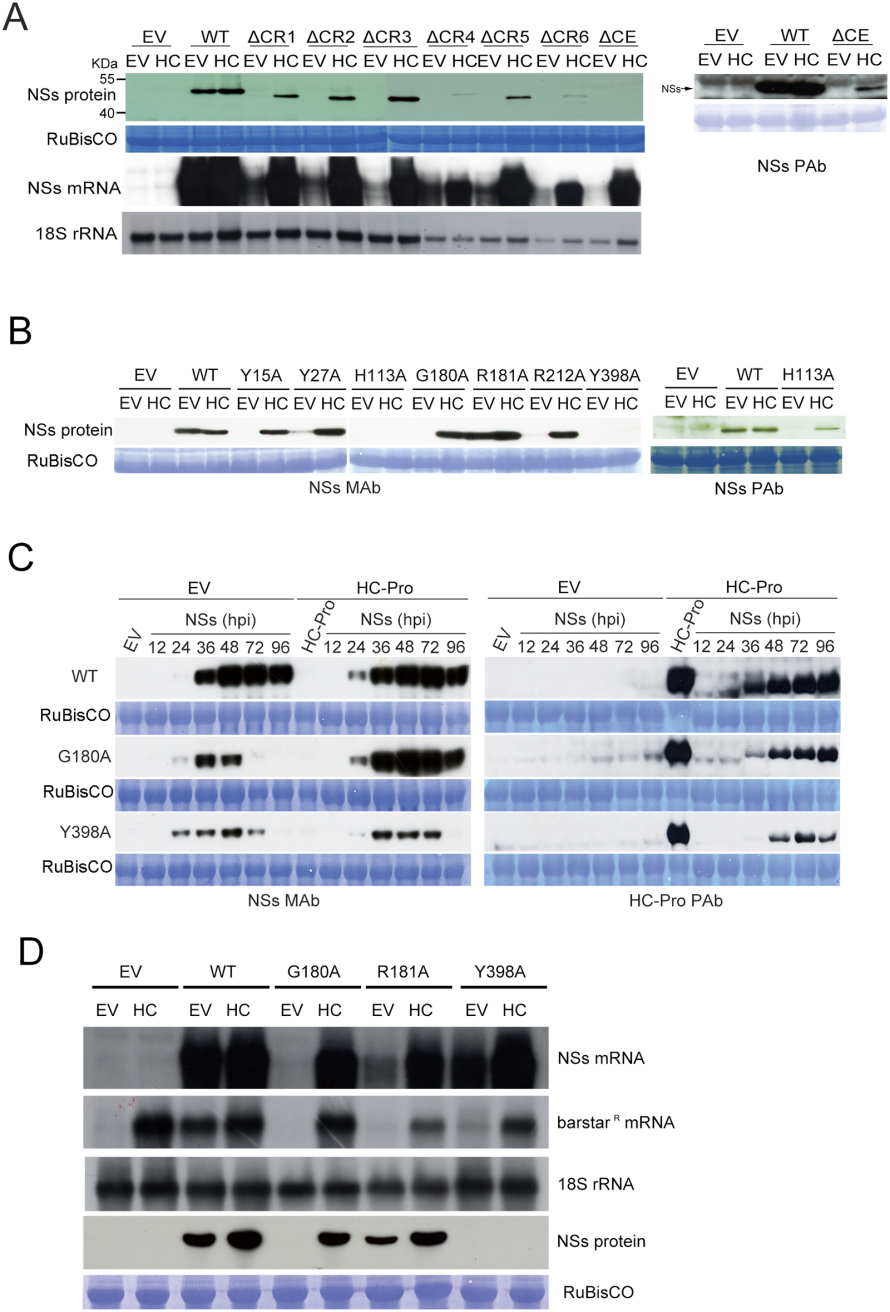
Expression levels of deleted and point-mutated NSs proteins analyzed by co-infiltration with potyviral suppressor HC-Pro. **(A)** Expression levels of protein and mRNA of deleted NSs mutants, following co-infiltration of the empty vector (EV) or HC-Pro (HC), detected at 4 day post agroinfiltration (dpa) by anti-NSs MAb (left panel) or PAb (right panel) and α-**32**P labeled NSs-probe, respectively. **(B)** Expression levels of point-mutated NSs proteins, following co-infiltration with the empty vector or HC-Pro, detected at 4 dpa by anti-NSs MAb (left panel) or PAb (right panel). **(C)** Time-course detection of the protein expression levels of the NSs mutants, G180A and Y398A, at different hours post infiltration (hpi) with EV or HC, detected by anti-NSs MAb (left panel) or anti-HC-Pro PAb (right panel). **(D)** Expression levels of G180A, R181A and Y398A NSs proteins, mRNA and barstar^R^ mRNA following co-infiltration of EV or HC construct, detected at 4 dpa. Coomassie Blue stained-RuBisCO proteins or 18S rRNA were used as loading controls.

In order to find out the possible cause for non-expression or lower expression of the deleted and point-mutated NSs mutants described above, each of them was co-infiltrated with the *Turnip mosaic virus* (TuMV) silencing suppressor HC-Pro. Our results showed that the accumulation of the mRNAs and proteins of ΔCR1- ΔCR6 and ΔCE could be rescued by HC-Pro, suggesting that the loss of RSS function of all deleted proteins is due to RNA silencing-mediated mRNA degradation that ultimately results in lack of protein expression (Fig 3A, upper panel).

Similar to the deletion mutants, when point mutants of Y15A, Y27A, H113A, G180A, R212A or Y398A were co-infiltrated with HC-Pro, all of them were also rescued, except for Y398A, suggesting that the loss of the RSS function of the mutants is also due to RNA silencing and lack of the protein expression (Fig 3A, lower panel). Since the Y398A protein could not be rescued by HC-Pro, this implies that the lack of protein expression of Y398A is not due to mRNA degradation.

In addition, in order to know the translation efficiency of mutated NSs protein, time-course experiment was carried out to detect the protein expression levels. The G180A or Y398Aproteins were expressed at levels even higher than the WT NSs protein during the early phase (24 hpi) following agroinfiltration (Fig 3C). However, G180A and Y398A protein expression levels decreased after 72 hpi. Interestingly, G180A protein expression level was rescued by HC-Pro, but not Y398A (Fig 3B). Similar to G180A, the protein expression levels of Y15A, Y27A, H113A, and R212A were also rescued during the same time course study (data not shown). The R181A mutant proteins were expressed at high levels when co-infiltrated with EV or HC-Pro, similar to that of the WT NSs. Our data indicate that the protein expression levels of all deleted or point-mutated NSs proteins, except for R181A and Y398A, were significantly enhanced by HC-Pro which apparently stabilizes the accumulation of the mutant mRNAs for protein expression through the RSS function of HC-Pro.

Overall, the seven point-mutated NSs proteins can be classified into three types: (1) Y15A, H113A, G180A and R212A lost RSS function and their proteins were undetectable, however, co-infiltration of HC-Pro rescued the normal accumulation of proteins; (2) the proteins of Y27A and R181A were detectable and their protein expression were enhanced by HC-Pro, but their RSS function was diminished; (3) Y398A lost RSS function and its protein was not detectable and not rescued by HC-Pro.

### Y398 residue is important for NSs protein stability, not for mRNA stability

Further experiments were conducted to analyze whether the accumulation of mRNAs of the point-mutated NSs mutants can be rescued by HC-Pro. Again, G180A, R181A and Y398A mutants were selected as representatives. They were co-expressed with the HC-Pro and the accumulation of their mRNAs and proteins was analyzed at 4 dpa. Without HC-Pro co-expression, the level of NSs mRNA accumulation of G180A was not detectable and the proteins of the G180A and Y398A mutants were not detected; and R181A showed a lower level of protein accumulation compared to that of the WT (Fig 3D). The barstar^R^ gene within the binary vector (pBCo), as an internal control in agroinfiltration, also showed lower or undetectable mRNA accumulation. The mRNA of barstar^R^ was therefore also silenced. However, its mRNA accumulation was restored only in the presence of the WT NSs protein but not the mutants of G180A, R181A and Y398A (Fig 3D). Thus, our results indicate that the WT NSs protein can protect both its own mRNA and the heterologous barstar^R^ mRNA through RNA silencing suppression, which was abolished or impaired by the three mutants tested.

During HC-Pro co-expression, although the mRNA accumulation of G180A, R181A and Y398A increased to high levels, only the proteins of G180A and R181A were subsequently enhanced (Fig 3D). The Y398A protein was not detected when co-infiltrated with the HC-Pro construct, even when its mRNA level was comparable to that of the WT NSs mRNA (Fig 3D).

Taken together, barstar^R^, G180A and R181A mRNAs were not detectable or barely detectable, but their expression could be rescued by HC-Pro, resulting in high levels of G180A and R181A protein accumulation. However, Y398A protein cannot be rescued by HC-Pro even when the accumulation of Y398A mRNA was enhanced to the level similar to that of the WT NSs. This result indicates that the Y398 residue is important for NSs protein stability, not for stabilizing its own mRNA.

### Protein expression and RNA silencing suppression capability of Y398A and R181A mutants

**Fig 4:**
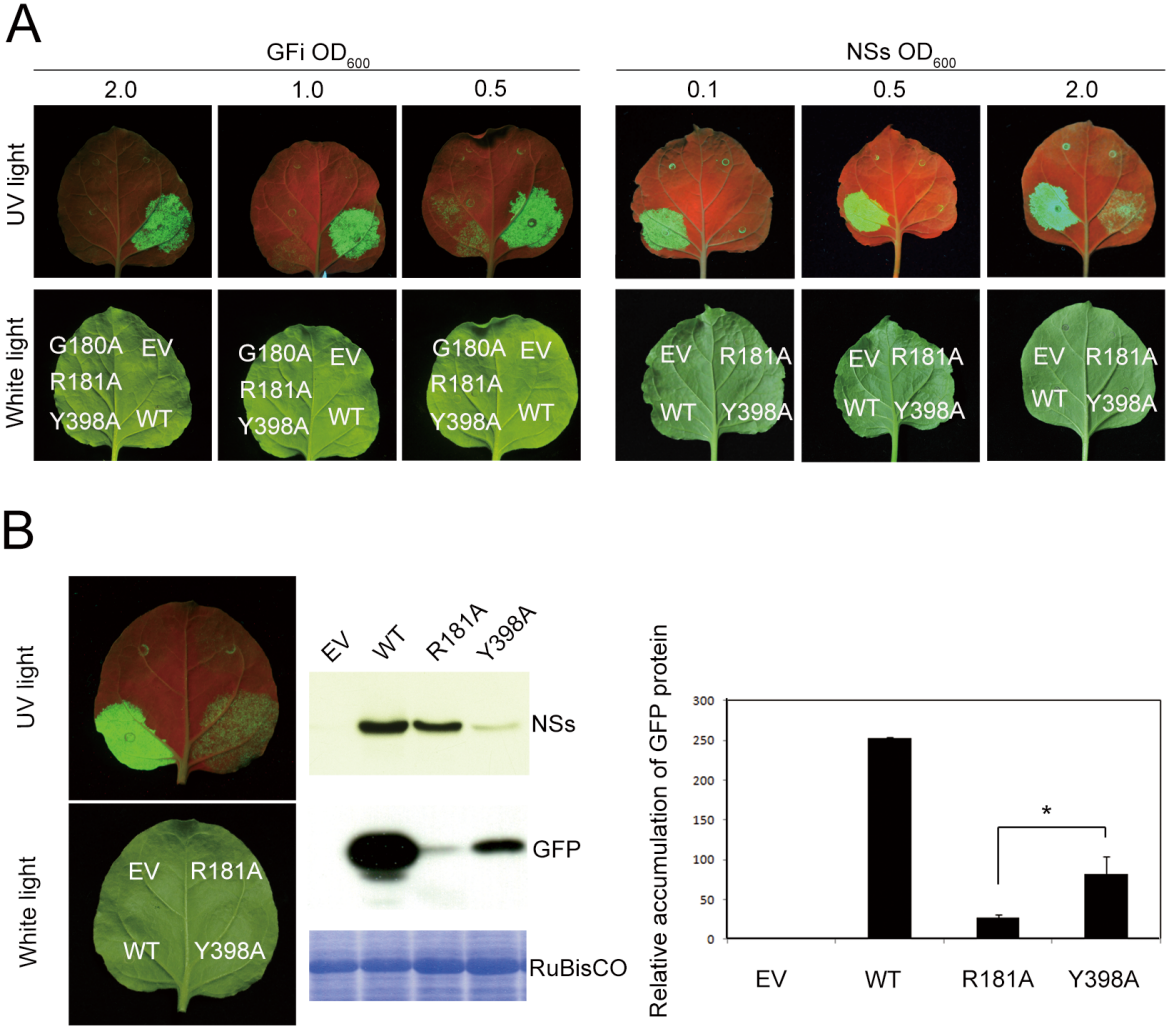
Analysis of RNA silencing suppression capability of mutated R181A and Y398A NSs proteins. **(A)** GFP intensity at leaf areas co-infiltrated with *Agrobacterium* strain carrying individual point-mutated NSs constructs and the strains separately carrying pBA-GFP and pBA-GFi constructs, recorded under white light or UV light illumination at 4 days post agroinfiltration (dpa). The concentrations of *Agrobacterium* culture carrying pBA-GFi (left panel) or individual NSs (right panel) constructs are shown on the top of each panel. The concentrations of *Agrobacterium* carrying pBA-GFP and NSs constructs (left panel) or pBA-GFP and pBA-GFi constructs (right panel) were adjusted to OD_600_ = 1.0. **(B)** Left panel: silencing suppression analyzed at 4 dpa. The relative proportions of *Agrobacterium* was 1:0.5:2 for GFP:GFi:NSs. Middle panel: western blotting was conducted for detection of mutated NSs protein and GFP protein from the leaf areas co-infiltrated with *Agrobacterium* cultures carrying GFP, hairpin GFP and individual NSs constructs. Total protein was extracted at 4 dpa. Coomassie blue stained RuBisCO proteins were used as loading controls. Right panel: The number indicates the relative accumulation of GFP co-infiltrated with individual NSs constructs, as quantified by Kodak image system 4000MM software. Statistically significant difference is indicated by “*”(n = 3, *P* = 0.012 < 0.05.).

In order to further analyze whether the loss of RSS function of point-mutated proteins is due to protein instability or direct dysfunction, the G180A, R181A and Y398A were again chosen as representatives for further assays. The concentration of *A*. *tumefaciens* carrying pBA-GFi was reduced to induce weaker RNA silencing activity. The GFP fluorescence after co-infiltration with mutated NSs constructs (OD_600_ 1.0) of R181A and Y398A was detectable at the concentration of *A*. *tumefaciens* carrying pBA-GFi at OD_600_ 0.5, whereas no fluorescence, was noticed whereas when pBA-GFi was at OD_600_1.0 or 2.0 (Fig 4A, left panel). These results indicate that RNA silencing activity was blocked by R181A and Y398A in a dosage dependent manner. Interestingly, RNA silencing activity was not inhibited by G180A, even at lower RNA silencing strength (pBA-GFi at OD_600_ 0.5), indicating that G180A lost RSS function completely and there was no dosage effect. In contrast, we also changed the concentrations of the cultures of *A*. *tumefaciens* carrying the WT, R181 or Y398A NSs construct. The GFP fluorescence was enhanced when the concentration of *A*. *tumefaciens* carrying Y398A was at OD_600_ 2.0 but not when the R181A mutant was used (Fig 4A, right panel).

Furthermore, we compared Y398A mutant with the R181A mutant that has impaired RNA silencing functional motif. Interestingly, the GFP fluorescence was not significantly enhanced in R181A-infiltrated leaf tissue, even its NSs protein amount was much higher than that of Y398A (Fig 4B), indicating that Y398A protein has higher RNA silencing suppression activity than R181A.

Thus, our data indicated that the loss of RSS function by G180A is due to its direct dysfunction, whereas the effect of R181A mutation is through partial impairment of the function. In addition, the weaker RSS function of Y398A mutant than the WT NSs protein is solely due to its poor stability, not as a result of the direct dysfunction of its RNA silencing suppression ability.

### The benzene ring of Y398 is essential for NSs protein stability and pathogenicty

**Fig 5:**
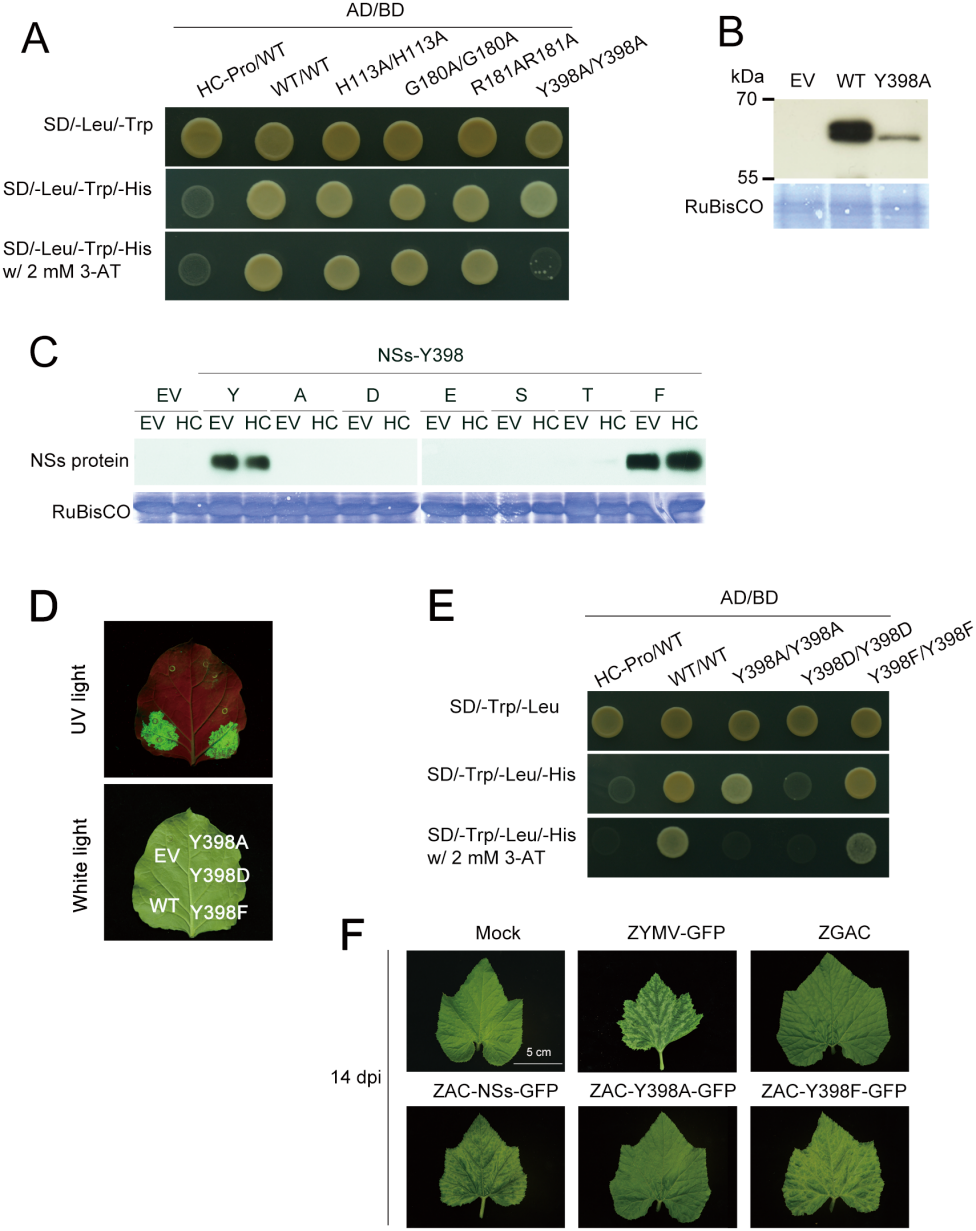
Characterization of Y398 on NSs self-interaction and protein stability. **(A)** Self-interaction analysis of NSs and mutants protein. Each co-transformed yeast cells grown on SD medium lacking Histidine (His), Leucine (Leu) and Tryptophan (Trp) without or with 2 mM of 3-AT. AD: activation domain, BD: binding domain. **(B)** Western blotting for detection of NSs protein expressed in yeast cells which were transformed with wild type NSs or Y398A construct. **(C)** Western blotting for detection of individual point-mutated NSs constructs, including wild type Y, mutated A, D, E, S, T, and F residues at the aa position 398 of NSs protein, co-infiltrated with empty vector (EV) or HC-Pro (HC-Pro) construct at 4 days post-agroinfiltration (dpa). Anti-NSs MAb was used for detecting NSs protein. Coomassie blue stained RuBisCO proteins were used as loading controls. **(D)** The RNA silencing suppression function of mutated NSs, Y398A, Y398D and Y398F, analyzed at 4 dpa. The relative proportions of *Agrobacterium* was 1:0.5:1 for GFP:GFi:NSs. **(E)** The self-interaction of mutated NSs, Y398A, Y398D and Y398F were examined by yeast two hybrid analysis. **(F)** Symptoms on squash plants after inoculation of individual ZYMV recombinants carrying Y398A or Y398F at 14 days post-infection.

Previous studies reported that other suppressors, p19 and 2b, form dimer which is important for its RSS function [52,53]. NSs protein has been reported to form inclusion body in cytoplasm [35], and may form dimer or oligomer [37]. We therefore examined the ability of the NSs point-mutated mutants to self-interact (Fig 5A).

The motif of aa 397–400 was predicted to be a *β*-sheet using POLVIEW-2D website (http://polyview.cchmc.org/). We compared the protein self-interaction of Y398A and other mutants of H113A, G180A and R181A by yeast two hybrid (Y2H) analysis. The H113A, G180A, R181A and Y398A mutants were able to self-interact. However, the strength of Y398A self-interaction was decreased when the competitive inhibitor of *HIS3* gene product, 3-AT, was increased (Fig 5A). Notably, the protein expression level of Y398A was much lower than WT NSs (Fig 5B). The data reveal that weaker self-interaction of Y398A in Y2H analysis was due to lower protein accumulation. This suggests that Y398A protein instability is due to incorrect protein folding and mutation dose not directly affects self-interaction.

Consequently, we assumed there potential factors that determine Y398A protein stability, one being phosphorlyation on the hydroxyl group of tyrosine (Y), another being the benzene structure of tyrosine. Therefore, the Y398 residue were substituted for other amino acids, including aspartic acid (D), glutamic acid (E), phenylalanine (F), serine (S) and threonine (T) s. The amino acids, D, E, S and T mimic phosphorlyation on Y398, whereas F mimics the benzene structure on Y398 but without a hydroxyl group. We found that only Y398F mutant showed high protein expression in *N*. *benthamiana* plants by agroinflitration at 4 dpa (Fig 5C). In addition, the RSS function and the strength of self-interaction of Y398F was restored (Fig 5D and 5E). This indicates that the benzene ring of tyrosine (Y) or phenylalanine (F) is important for NSs protein stability.

ZGAC carrying GFP induces attenuated symptoms on squash plants [47], and is therefore as an ideal viral vector for analyzing the pathogenicity of a heterologous viral factor. The plants infected with the WT ZYMV carrying GFP showed typical symptoms of yellowing, mosaic and leaf destruction at14 days post-infection (dpi), while the plants infected with ZGAC showed highly attenuated symptoms of transient mottling and then recovered to no conspicuous symptoms in the upper leaves which were similar to those of healthy plants (Fig 5F). The ZGAC recombinant viruses carrying the WT NSs gene (ZAC-NSs-GFP) induced severe symptoms of severe mosaic and leaf distortion on squash without yellowing (Fig 5F), indicating that the NSs protein can complement the mutations in the HC-Pro of ZGAC and restore its pathogenicity on squash. When ZAC-Y398F was analyzed for pathogenicity, the result revealed that ZAC-Y398F caused symptoms similar to that caused by ZAC-NSs on squash (Fig 5F), indicating that the hydroxyl group does not affect pathogenicity.

Taken together, Y398 does not affect protein self-interaction, but the benzene of Y398 is essential for NSs protein stability and thus indirectly affects RSS function and pathogenicity.

### Mutated NSs proteins of Y15, Y27, H113, G180, R181, R212 and Y398A affect pathogenicity in squash plants as assayed by mild ZYMV GAC vector

The tospoviral NSs gene and its potyviral counterpart HC-Pro gene have been reported to be the main pathogenicity factors of the respective viral genera [15,35]. Here, the NSs amino acids found to be critical for RSS function were further investigated for their roles in pathogenicity.

**Fig 6:**
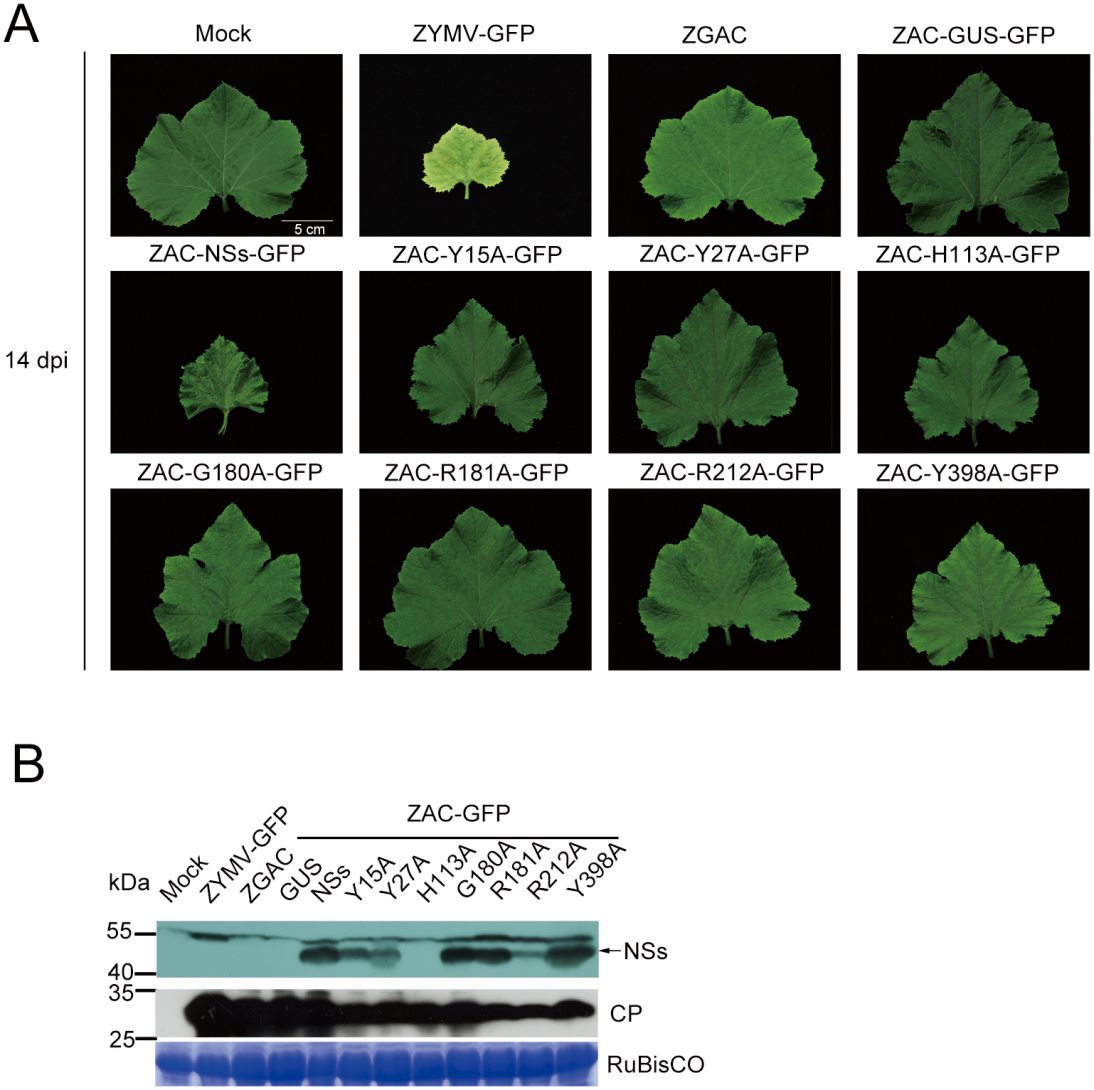
Symptoms on squash plants after inoculation with *Zucchini yellow mosaic virus* (ZYMV) recombinant viruses carrying different point-mutated NSs proteins. **(A)** Symptoms on squash plants inoculated with individual ZYMV recombinants at 14 days post-infection (dpi). **(B)** Detection of mutated NSs proteins expressed by individual ZYMV recombinants at 14 dpi using anti-NSs MAb or anti-ZYMV CP PAb. Coomassie blue-stained RuBisCO protein was used as loading controls.

After analysis by ZGAC vector, we found that the WT NSs protein can complement the mutations in HC-Pro of ZGAC and restore its pathogenicity on squash plants (Figs 5F and 6A). Interestingly, the ZGAC recombinants carrying individual point-mutated NSs proteins of Y15A, Y27A, H113A, G180A, R181A, R212A, and Y398A induced attenuated symptoms on squash at 14 dpi, much milder than that induced by the WT NSs protein (Fig 6A).

In addition, mutated NSs proteins, except for H113A, were detected by Western blotting using anti-NSs MAb (Fig 6B) as well as anti-NSs PAb, which was generated using the antigen from ZYMV-NSs recombinant-infected squash [45] and had heavy strong background on squash extracts, rendering the detection of H113A protein difficult (data not shown). In order to check the fidelity of the mutated proteins, the NSs ORF region in each ZYMV recombinant was amplified by RT-PCR and sequenced. The results indicated that each mutated NSs ORF was correctively sustained and expressed in ZYMV recombinants.

Therefore, our results indicated that the amino acids Y15, Y27, H113, G180, R181, R212 and Y398 of the NSs protein are not only essential for RSS function, but are also important for pathogenicity of the NSs protein on squash plants. Furthermore, mutation of Y398 residue also affects the NSs pathogenicity although to a lesser degree than the mutations of other critical amino acids.

## Discussion

In this study, we identified six highly conserved regions (CRs) and the common epitope NScon in the NSs proteins of WSMoV, an Asia type tospovirus, that are indispensable for its RSS function. In addition, alanine mutagenesis of the residues Y15 and Y27 (located in CR1), H113 (located in NSscon), G180, R181 and R212 (located in CR3), Y398 (located in CR6) negatively affected the RSS function of the protein. Moreover, our results from expression analyses of the mutant proteins with ZYMV mild vector in squash plants revealed that all seven residues are also critical for viral pathogenicity.

Furthermore, when we further aligned 25 newly identified tospoviruses NSs protein sequences currently available in GeneBank, conserved motifs were fully aligned except for Y398 where the corresponding aa in BeNMV is a Phenylalanine (F). The alignment data match our assumption that the benzene of Y398 in WSMoV NSs is conserved and important for its functions.

### The criticality of Y15, Y27 and R212 for RNA silencing suppression

Previous reports have identified several functional motifs of Euro-America type tospoviral NSs proteins important for its RSS function [43,44]. In this study, we identified seven aa residues of Asia-type WSMoV NSs protein critical for the RNA silencing suppression of WSMoV NSs protein. Among them Y15, Y27, G180, R181 and R212 are located at similar positions and have the structures of the reported functional motifs of TSWV NSs protein (W17, Y30, G181, K182 and R211) important for the RSS function [43,44].

Being an aromatic amino acid, the Y15 of WSMoV NSs may possibly play a similar role to W17 of TSWV NSs protein which is suggested as the aromatic residue of the putative AGO binding motif (GW/WG) [43]. However, in WSMoV NSs protein, the context containing Y15 does not bear any resemblance to the canonical GW/WG motif. Furthermore, our result of Y2H did not show interaction between WSMoV NSs protein and AGO1 proteins (data not shown). It is possible that the aromatic amino acid, Y15 and Y27, or positively charged amino acid, R212, may be responsible for RNA binding [54].

### The criticality of G180 and R181 for ATPase and RNA binding activities

The presently described G180 and R181 residues from the CR3 of WSMoV are the constituents of the GKV/T motif, which is part of a Walker A motif (GXXXXXGKT) [44]responsible for ATPase activity and RNA binding [39]. Recently, a study indicated that GBNV NSs protein has DNA helicase activity and the Walker A motif is essential for the helicase function but not for RSS function [55]. Hence, we suggest that the G180 and R181 residues of WSMoV NSs protein also have ATPase, RNA binding and DNA helicase activities that are not similar to the role in RSS function of GBNV NSs protein. In addition, we found that G180 and R181 residues in the Walker A motif are also responsible for pathogenicity on squash.

### The common epitope NSscon is an essential region for binding to host factor for the RNA silencing suppression function

As shown by the present results, CE region, especially residue H113 of NSs protein was essential for its RNA silencing suppression ability. The CE is highly conserved among Asia-type tospoviruses [45]. When the core nine aa residues (nss) of the CE was deleted or the H113 at the center of the nss was mutated, the MAb does not react with the modified CE [45,46] (Fig 2). Our findings suggest that the CE may be a region that interacts with a host or a viral factor vai its unique feature, similar to its interaction with the NSscon-recognizing MAb [45,46]. The mRNA and protein of H113A were not detectable when transiently expressed by agro-infiltration (Fig 2C). However, when H113A construct was co-infiltrated with HC-Pro construct, the steady state level of the mutant protein was rescued to the level similar to that of the WT NSs protein (Fig 3B). Our results indicate that the loss of RSS function of H113A renders its own transcript susceptible to PTGS-mediated degradation and thus its translation is abolished.

### Tyrosine 398 is needed for the protein stability of NSs protein

The substitution of tyrosine (Y) 398 with an alanine residue abolished the RSS function of NSs protein and rendered the protein undetectable. Unlike H113A mutant, the steady state level of Y398A mutant protein was not rescued by HC-Pro, though the Y398A transcript was rescued to a level similar to that of the WT NSs (Fig 3). Our results indicate that Y398A mutation does not affect the stability of NSs mRNA but renders the translated protein unstable.

Both the WT NSs protein with Y398 and its point mutant with F398 were equally proficient in RSS function. The aromatic residues, Y, F and W found in *β*-sheets may stabilize proteins when hidden in the protein or mediate dimerization/ oligomerization and aggregation when present in the protein surface. Secondary structural analysis of NSs revealed the presence of Y398 in a four hydrophobic aa *β*-sheet ^397^IYFL^400^, which appears to be a surface motif involved in NSs protein aggregation and/or self-interaction. Our result of Y2H analysis indicates that Y398 is not involved in self-interaction of NSs protein (Fig 5A). In addition, the H113, G180 and R181 were also not essential for self-interaction, implying that these motifs of NSs protein required for RSS function are not related to self-interaction.

NSs protein aggregates in the plant cells as filament inclusion body in cytoplasm [35]. Therefore, we assumed aggregation of NSs protein may be important for its stability and functions, including RSS function and pathogenicity. Prevention of aggregation by Y398A mutation may result in depletion of NSs protein and consequently abolish NSs protein-mediated RSS function. Nevertheless, the actual role of NSs inclusion body, which may not present in different hosts, remains to be further investigated.

On the other hand, the degradation of the Y398A mutated protein may be proteasome-independent, as suggested by the failure of the proteasome inhibitor MG132 to rescue the accumulation of Y398A mutant protein to the WT level after agroinfiltration in plant (data not shown).

### ZYMV GAC is an excellent vector for investigating the pathogenicity determinant of a plant virus in cucurbitaceous plants

The subversion of normal plant development and physiology through binding and interference with plant miRNAs pathway by viral RSS is the major cause of viral disease symptoms in plants [21,23,56,57]. Furthermore, our recent studies provided the molecular evidence on the roles of the mutations of ZYMV or TuMV HC-Pro in miRNA and siRNA pathways in host plants [21,58]. Here, the pathogenicity of the essential aa residues (Y15, Y27, H113, G180, R181, R212 and Y398) were analyzed by ZYMV GAC vector [47]. We showed the ZAC-NSs-GFP cause severe symptom similar to that of the WT ZYMV (Figs 5F and 6A). Our results indicate that NSs can complement the HC-Pro mutations that result in abolishment of miRNA interference [21,58], and thus restored the severe type symptoms. Apparently, NSs protein and HC-Pro interfere with common miRNA pathways [37]. Together, we conclude that NSs protein is important for pathogenicity of WSMoV. However, since we did not test other WSMoV proteins using the ZYMV mild vector, the possibility that other WSMoV proteins may play a similar role like NSs protein cannot be excluded.

By our unique ZYMV GAC vector, we found that the silencing suppression function of tospoviral NSs gene correlates with viral pathogenicity. However, symptom development by virus infection may be variable under different environmental conditions, especially temperature. In our previous observation [47], the differences in symptoms between ZYMV or ZAC mild strain infected plants are not affected under different conditions. Thus, we tried to conduct tests under temperature controlled greenhouse for more homogenous comparison of recombinants’ symptoms.

Together, our results indicate that ZYMV GAC is an excellent vector for investigating the pathogenicity determinant of a plant virus in cucurbitaceous plants.

## Conclusions

In this study we have identified critical aa residues in various conserved regions of WSMoV NSs protein responsible for the RSS function. These essential residues can be classified into three types: (1) affecting RNA silencing functional motif completely and thus resulting in instability of NSs mRNA: all deletion mutants and point-mutated mutants of Y15A, Y27A, H113A, G180A and R212A; (2) partially impairing RNA silencing functional motif: Y27A and R181A; and (3) affecting NSs protein stability: Y398A. In particular, we uncovered that two highly conserved amino acids, H113 at nss (^109^KFTMHNQ^117^) and Y398 at C-terminal *β*-sheet motif (^397^IYFL^400^) are important for NSs RNA silencing suppression and pathogenicity by affecting its own mRNA or protein stability, respectively. By ZYMV mild vector, we also show evidence that the RSS function of NSs protein correlates to viral pathogenicity. Thus, our results provide valuable information for RNA silencing suppression and pathogenicity of tospovirus, and insight for modulating the host-virus interaction for virus control.

## Supporting Information

S1 Fig Construction of p35SZAC-DC-nGFP for analyzing pathogenicity. NOS-T: the terminator of nopaline synthase gene; 35S-P: Cauliflower mosaic virus 35S promoter; DC: destination cassette; GFP: green fluorescent protein; P1, HC-Pro, P3, CIP, NIa-VPg, NIa-Pro, NIb and CP genes of ZYMV are shown.
(TIF)

S1 Material and Methods Construction of NSs gene in ZYMV viral vector for analyzing pathogenicity. (DOCX)

S1 Table Olignucleotide primers used in this study. ^a^ Underlined bases indicate restriction sites (RE), bolded bases represent mutated nucleotides and lower-case letters denote non-viral sequences. ^b^ Polarity to *watermelon silver mottle virus* NSs coding sequence.
(PDF)
