# Supplementary figures and images for: Two Novel Motifs of Watermelon Silver Mottle Virus NSs Protein Are Responsible for RNA Silencing Suppression and Pathogenicity
Source: PLoS One. 2015 May 20;10(5):e0126161. doi: 10.1371/journal.pone.0126161 (PMC4439075; doi:10.1371/journal.pone.0126161)

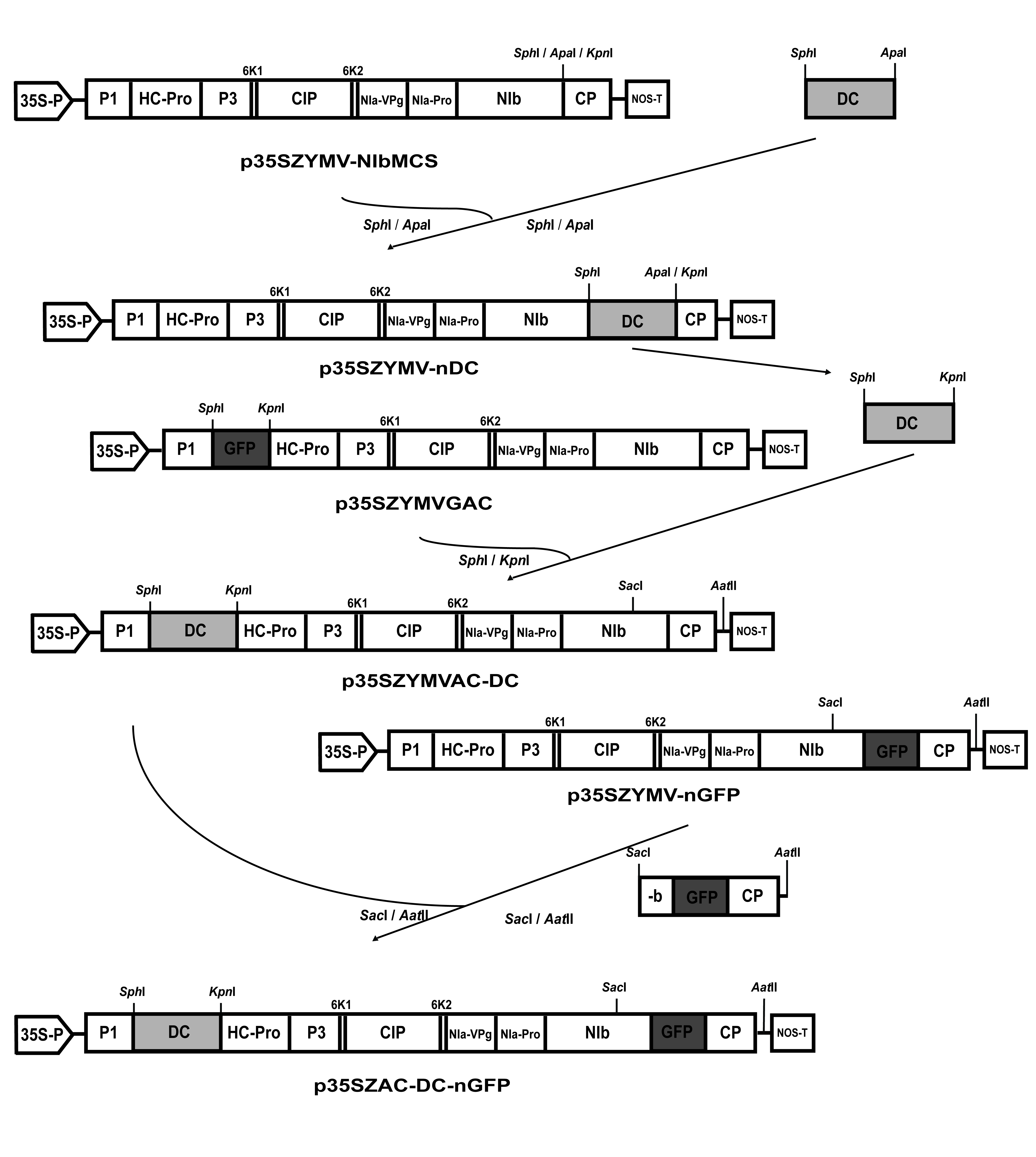

Supplement: S1 Fig — NOS-T: the terminator of nopaline synthase gene; 35S-P: Cauliflower mosaic virus 35S promoter; DC: destination cassette; GFP: green fluorescent protein; P1, HC-Pro, P3, CIP, NIa-VPg, NIa-Pro, NIb and CP genes of ZYMV are shown. (TIF) [file pone.0126161.s001.tif]
