# Supplementary material for: Two Novel Motifs of Watermelon Silver Mottle Virus NSs Protein Are Responsible for RNA Silencing Suppression and Pathogenicity
Source: PLoS One. 2015 May 20;10(5):e0126161. doi: 10.1371/journal.pone.0126161 (PMC4439075; doi:10.1371/journal.pone.0126161)
Supplement: S1 Material and Methods — (DOCX) [file pone.0126161.s002.docx]

**S1 Material and Methods**

**Construction of NSs gene in ZYMV viral vector for analyzing pathogenicity**

In order to construct ZYMV viral vector as a Gateway destination vector for simpler and faster cloning, plasmid pBCo-DC-myc [21] was used as a template for PCR. Primers used to amplify the coding sequence of the Gateway^®^ destination cassette were the 5'-end primer P-DCattR1-SphI and the 3'-end primer N-DCattR2-ApaI. The PCR products were cloned in pCR2.1-TOPO vector (Invitrogen, Carlsbad, CA, USA). The DNA fragment of the Gateway^®^ destination cassette was released from the pCR2.1-TOPO vector with restriction enzymes *Sph*I and *Apa*I. The GFP fragment was released from p35SZYMVGFPhis-3 [47] with restriction enzymes *Sph*I and *Kpn*I. The coding sequence of the Gateway destination cassette digested fragment was inserted into infectious clone p35SZYMVNIbMCS [47] to generate p35SZYMV-nDC. The Gateway destination cassette was released from p35SZYMV-nDC with restriction enzymes *Sph*I and *Kpn*I to replace the GFP fragment of p35SZYMVGAC (mild strain) to generate p35SZYMVAC-DC. At the final step, p35SZYMV-nGFP was digested with restriction enzymes *Aat*II and *Sac*I to exchange the corresponding portion of ZYMV cDNA sequence of p35SZYMVAC-DC to generate p35SZAC-DC-nGFP (S1 Fig.). These two ZYMV vectors carrying DC fragment were convenient to introduce DNA fragment into these ZYMV vector without restrict enzyme site.

The role for pathogenicity of the highly conserved aa motifs of the NSs protein was analyzed on squash plants by ZYMV vector. The NSs-Y15A ORF was amplified by PCR with the reverse primer N-WNSs-nonstop and forward primer P-caccWNSs from pBCo-NSs-Y15A. The amplified fragment was cloned in pENTR/D vector (Invitrogen, Carlsbad, CA, USA) to generate pENTR/WNSs-Y15A. The pENTR/WNSs-Y15A was transferred to p35SZAC-DC-nGFP by LR Clonase enzyme to generate p35SZAC-Y15A-GFP. The wild type NSs gene and other mutated NSs variants were cloned in p35SZAC-DC-nGFP by the similar procedure.
