# Supplementary material for: Two Novel Motifs of Watermelon Silver Mottle Virus NSs Protein Are Responsible for RNA Silencing Suppression and Pathogenicity
Source: PLoS One. 2015 May 20;10(5):e0126161. doi: 10.1371/journal.pone.0126161 (PMC4439075; doi:10.1371/journal.pone.0126161)
Supplement: S1 Table — a Underlined bases indicate restriction sites (RE), bolded bases represent mutated nucleotides and lower-case letters denote non-viral sequences. b Polarity to watermelon silver mottle virus NSs coding sequence. (PDF) [file pone.0126161.s003.pdf]

S1 Table Olignucleotide primers used in this study

Name	Sequence (5' to 3')[a]	Polarity[b]	RE site[a]
**Binary vector**			
P-caccWNSs	caccATGTCTACTGCAAAGAAT	+	
N-WNSs nonstop	TTCTGCTTTCACAACAAA	-	
N-WNSs	TTTGTTGTGAAAGCAGAATAA	-	
P-WNSs-*Asc*I	ggcgcgccATGTCTACTGCAAAGAATGCTG	+	*Asc*I
P-2stop-NSs-*Asc*I	ATGTGATGATCTACTGCAAAGAATGCTG		*Asc*I
N-WNSs-*Bam*HI	cgggatccTTATTCTGCTTTCAC	-	*Bam*HI
**Deletion mutation**			
P-WNSs-ΔCR1	ggcgcgccATGTCTGTATTCACTGGAGAAG	+	*Asc*I
P-WNSs-ΔCR2	AACGATTTGGGCAGGATAACATTCTGCGAGCATGA	+	
P-WNSs-ΔCR3	AATGGAGATCTTACCTTGCTATCTACATCAACAGT	+	
P-WNSs-ΔCR4	ATCAGTTTTTACGCAGTGGTTACAAAGACTGTTGA	+	
P-WNSs-ΔCR5	GATTTCTGTGATAGTCTCCACAATGATTATACAGA		
P-ΔWNSscon	GGACTAGACATAACAATGCACAATCAAATCTTTAATCCA	+	
N-WNSs-ΔCR2	CTCGCAGAATGTTATCCTGCCCAAATCGTTGATGC	-	
N-WNSs-ΔCR3	TGATGTAGATAGCAAGGTAAGATCTCCATTGGTAG	-	
N-WNSs-ΔCR4	AGTCTTTGTAACCACTGCGTAAAAACTGATCATTC	-	
N-WNSs-ΔCR5	ATAATCATTGTGGAGACTATCACAGAAATCAACAA	-	
N-WNSs-ΔCR6	ggatccTTATTCTGAATCTACCTGGTAATTC	-	*Bam*HI
N-ΔWNSscon	GAAAAATTTGGACTAGACATAACAATGCACAATCAAAT		
**Point-mutation**			
P-Y15A	GAATTCATCAAAAGT**GC**TGGCACAAAAGAC	+	
N-Y15A	GTCTTTTGTGCCA**GC**ACTTTTGATGAATTC	-	
P-G16A	GAATTCATCAAAAGTTATG**C**CACAAAAG	+	
N-G16A	CTTTTGTG**G**CATAACTTTTGATGAATTC	-	
P-D25A	GAGCAGTTAATG**C**TTGCTACTCTGTATTC	+	
N-D25A	GAATACAGAGTAGCAA**G**CATTAACTGCTC	-	
P-Y27A	GCAGTTAATGATTGC**GC**CTCTGTATTC	+	
N-Y27A	GAATACAGAG**GC**GCAATCATTAACTGC	-	
P-E61A/D62A	GGGCAGGAATG**C**AG**C**TGTCAAAATCC	+	
N-E61A/D62A	GGATTTTGACA**G**CT**G**CATTCCTGCCC	-	
P-K64A	GAAGATGTC**GC**AATCCATGAGGCTG	+	
N-K64A	CAGCCTCATGGATT**GC**GACATCTTC	-	
P-E67A/E69A	CAAAATCCATG**C**GGCTG**C**AGTTGTTG	+	
N-E67A/E69A	CAACAACT**G**CAGCC**G**CATGGATTTTG	-	
P-D86A	GAAAAATTTGGACTAG**C**CATAACATTC	+	
N-D86A	GAATGTTATG**G**CTAGTCCAAATTTTTC	-	
P-C108A	ACAGGC**GCT**AAGTTCACAATGCACAATCAAATC	+	*Dde*I
N-C108A	TGCATTGTGAACTT**AGC**GCCTGTGTTCTTCACACC	-	*Dde*I
P-K109A	ACAGG**G**TGC**GCA**TTCACAATGCACAATCAAATC	+	*Dra*III
N-K109A	TGCATTGTGAA**TGC**GCA**C**CCTGTGTTCTTCACACC	-	*Dra*III
P-H113A	CAAGTTCACAATG**GC**CAATCAAATC	+	
N-H113A	GATTTGATTG**GC**CATTGTGAACTTG	-	
P-G180A	GTTTTCTGTAATGG**C**AAGAACAACATCTTAC	+	
N-G180A	GTAAGATGTTGTTCTT**G**CCATTACAGAAAAC	-	

P-R181A	CTGTAATGGGA**GC**AACAACATCTTAC	+	
N-R181A	GTAAGATGTTGTT**GC**TCCCATTACAG	-	
P-K199A	CTTGTCAGTT**GC**GCAAAAATGCTTTTC	+	
N-K199A	GAAAAGCATTTTTGC**GC**AACTGACAAG	-	
P-R212A	CCCTACCAAC**GC**ATTGCTATCTAC	+	
N-R212A	GTAGATAGCAAT**GC**GTTGGTAGGG	-	
P-E291A	GACAACAGTAATG**C**GAGAACCACCCTTATC	+	
N-E291A	GATAAGGGTGGTTCTC**G**CATTACTGTTGTC	-	
P-R292A	GACAACAGTAATGAG**GC**AACCACCCTTATC	+	
N-R292A	GATAAGGGTGGTT**GC**CTCATTACTGTTGTC	-	
P-I342A	AATTACAATCAATTG**GC**TGTCAAGAATCTGTTAA	+	
N-I342A	TTAACAGATTCTTGACA**GC**CAATTGATTGTAATT	-	
P-K369A	CATAGTGTTC**GC**AATGTATGATAAAGAGC	+	
N- K369A	GCTCTTTATCATACATTGCGAACACTATG	-	
P-E374A	GTATGATAAAG**C**GCTCCACAATGATTATAC	+	
N-E374A	GTATAATCATTGTGGAGC**G**CTTTATCATAC	-	
P-Y398A	GAAGGGAATATT**GC**TTTCCTCTCAAAGAC	+	
N-Y398A	GTCTTTGAGAGGAAA**GC**AATATTCCCTTC	-	
P-T403A	CTCTCAAAG**G**CTCTTGAGGTTC	+	
N-T403A	GAACCTCAAGAG**C**CTTTGAGAG	-	
P-Y398D	CAGAAGGGAATATT**G**ATTTCCTCTCAAAGAC	+	
N-Y398D	GTCTTTGAGAGGAAAT**C**AATATTCCCTTCTG	-	
P-Y398E	GAAGGGAATATT**G**A**G**TTCCTCTCAAAG	+	
N-Y398E	CTTTGAGAGGAA**C**T**C**AATATTCCCTTC	-	
P-Y398F	CAGAAGGGAATATTT**T**TTTCCTCTCAAAGAC	+	
N-Y398F	GTCTTTGAGAGGAAA**A**AAATATTCCCTTCTG	-	
P-Y398S	GAAGGGAATATTT**C**TTTCCTCTCAAAG	+	
N-Y398S	CTTTGAGAGGAAA**G**AAATATTCCCTTC	-	
P-Y398T	GAAGGGAATATT**AC**TTTCCTCTCAAAG	+	
N-Y398T	CTTTGAGAGGAAA**GT**AATATTCCCTTC	-	
**mRNA stability**			
P-2XstopNSs-*Asc*I	ggcgcgccATGTAATAATCTGTATTCACTGGAGAAG	+	*Asc*I
P-TNSs	caccATGTCTTCAAGTGTTTATGA	+	
N-TNSsnonstop	TTATTTTGATCCTGAAGCACATGC	-	
**Viral vector expression**			
P-WNSs-*Nco*I	catgccatggcaATGTCTACTGCAAAG	+	*Nco*I
N-WNSs-*Nhe*I	gctagcTTCTGCTTTCACAACAAAG	-	*Nhe*I
P-DCattR1-*Sph*I	gcatgcACAAGTTTGTACAAAAAAGCTG	+	*Sph*I
N-DCattR2-ApaI	gggcccCACCACTTTGTACAAGAAAGC	-	*Apa*I

[a] Underlined bases indicate restriction sites (RE), bolded bases represent mutated nucleotides and lower-case letters denote non-viral sequences.

[b] Polarity to *watermelon silver mottle virus* NSs coding sequence
